# Epidemiology of Viral Hepatitis and Liver Diseases in India

**DOI:** 10.5005/iD-iournals-10018-1126

**Published:** 2015-01-06

**Authors:** Pradeep Bhaumik

**Affiliations:** Department of Gastroenterology, Agartala Government Medical College, Agartala, Tripura, India

**Keywords:** Hepatitis B virus, Hepatitis C virus, Prevalence, Tribal population, Blood donors.

## Abstract

Diverse Indian population provides an excellent opportunity to study the prevalence and feature of hepatitis virus for understanding viral evaluation and viral pathogenesis. India is in intermediate zone of pathogenesis of hepatitis B (2-5%), and India is having around 40 million hepatitis B virus (HBV) carriers. A wide variation in prevalence of hepatitis B is observed from region to region and community to community. The prevalence of HBV is higher among tribal population, than nontribal.

Population prevalence of hepatitis C virus (HCV) infection in India is 1%. Prevalence study among blood donors may reflect population prevalence. Hemodialysis increases the possibility of blood borne viral infection. Higher prevalence of HBV among tribal population is of paramount importance from public health point of view and hepatitis B vaccination.

**How to cite this article:**

Bhaumik P. Epidemiology of Viral Hepatitis and Liver Diseases in India. Euroasian J Hepato-Gastroenterol 2015;5(1):34-36.

## INTRODUCTION

Diverse Indian population provides an excellent opportunity to study the prevalence and features of hepatitis viruses for understanding viral evolution and disease pathogenesis. Though India has diverse community group, there is paucity of studies at community level. It has been observed that among aborigine groups, hepatitis B virus (HBV) prevalence is much higher than other people. However, all the aborigine groups and ethnic group have not been evaluated extensively. In this mini-review, we would try to accumulate the available data in the literature. Prevalence study among the voluntary blood donors has also been included in our database. However, it reflects only a section of people.

## HEPATITIS B IN COMMUNITY

Among the South-East Asian countries, India is in intermediate zone of prevalence (2 to 5%).^1^ National Center for Disease Control (NCDC), India, reported a 3.7% point prevalence, i.e. over 40 million HBV carriers in India.^2^ A wide variation in prevalence of hepatitis B is observed from region to region and community to community. Hepatitis B virus prevalence at community level in Tripura (North-East region of India) is 3.6% (95% CI 3.14-4.06), West Bengal 2.97%,^3^ Tamil Nadu 5.7% (95% CI 4.6-6.8),^4^ Northern India 2.1%.^5^

## HEPATITIS B AMONG TRIBAL POPULATION

The prevalence rate of HBV is higher among tribal than nontribal population. In a meta-analysis by Batham et al reported that the prevalence of HBV in tribal and non-tribal population is 15.9% (95% CI 11.4-20.4) and 2.4% (95% CI 2.2-2.7) respectively.^6^ The prevalence of HBV differs in different tribal community in India.

## HEPATITIS C IN INDIA

According to NCDC, the population prevalence of HCV infection in India is 1%.^2^ In a community-based study, Chowdhury A from West Bengal reported 0.87% of HCV prevalence.^7^ In Andhra Pradesh, 2.02% of HCV seropreva-lence has been found in Lambada community.^8^

## PREVALENCE OF HEPATITIS B AND C AMONG BLOOD DONORS IN INDIA

Prevalence of hepatitis B and C among voluntary blood donors (Table 1) in different parts of India has been shown in following Table:

**Table:** 

*Place*		*HBV (%)*		*HCV (%)*		*Reference*	
Tripura		1.2		0.109		Unpublished data	
Andhra Pradesh		1.41		0.84		Bhawani Y et al (2010)	
Ahmedabad		0.977		0.108		Shah N et al (2013)	
Bhopal		2.9		0.57		Sawke N et al (2010)	
Maharashtra		1.09		0.74		Purushottam A et al (2012)	
New Delhi		0.2		0.7		Pathak S et al (2013)	
New Delhi				1.57		Jain A et al (2003)	
Patiala				0.88		Bagga PK et al (2007)	
New Delhi		1.66		0.65		Gupta R et al (2011)	
Chandigarh		1.7		0.8		Kaur G et al (2010)	
Lucknow		1.67		0.49		Chandra T et al (2014)	
Uttar		1.5		0.8		Agrawal VK et al (2012)	
Pradesh							
Uttarakhand		1.2		0.9		Negi G et al (2014)	
East Delhi		1.8		0.5		Singh B et al (2004)	
Uttar		1.96		0.85		Chandra T et al (2009)	
Pradesh							
Kerala		1.5		0.4		Anjali H et al (2012)	
Kolkata		1.4		0.59		Karmakar PR et al (2014)	

## PREVALENCE OF HEPATITIS B AND C AMONG HEMODIALYSIS PATIENTS

It has been reported that hemodialysis increases the possibility of blood borne viral infection but the prevalence is variable from hemodialysis from center to center and also from region to region and country to country, and high-cost hemodialysis center *vs* low-cost hemodialysis center. It is not well understood whether this variability has got any relationship with the basic prevalence of the disease in the community.

In most of the study, HBV infection among hemodia-lysis patients was between 4 and 11% and HCV infection was between 8 and 12%.

In India, reported study of HBV and HCV infection among hemodialysis patient is variable. In Tripura, it is found that the prevalence of hepatitis B and C among hemodialysis patients is 7.3 and 12.1% respectively, and 1.2% patients were coinfected with both viruses.

Reddy et al have reported that, among hemodialysis patients, 5.9% were HCV-positive while 1.4% patients had HBV infection and 3.7% had coinfection with HBV and HCV.

We have reported that among the patients of chronic kidney disease, renal transplant or hemodialysis, HBV, HCV and coinfection of both viruses were 7, 46, and 37.1°% respectively.^9^

## CONCLUSION

It has been observed that prevalence of HBV at community level in India is highly variable with a higher prevalence in Tribal population than nontribal population. Higher prevalence of HBV among tribal population is of paramount importance from public health point of view and early intervention by hepatitis B vaccination will reduce the disease burden among tribal population in India. But, in hemodialysis patients, prevalence of HBV and HCV is much higher than community level. In a community where HBV and HCV infection prevalence is higher needs adoption of mass hepatitis B vaccination and further precautionary measures to reduce mortality and morbidity among chronic kidney disease patients. Prevalence among blood donors can be a ready reference of community status and impact of preventive measures.

**Table 1:** **Table 1:** Prevalence of hepatitis B among tribal groups of India

*State*		*Tribe*		*HBsAg (%)*		*Reference*	
Tripura		Chakma		11.41		Unpublished data	
		Reang		7.69			
		Noatia		6.09			
		Murasing		5.15			
Andaman and		Onges		31		Murhekar MV	
Nicobar Islands						et al (2000)	
		Nicobarese		23.3			
		Shompens		37.8			
		Jarawas		65.6		Murhekar MV	
						et al (2003)	
Madhya		Baiga		4.4		Reddy PH et al	
Pradesh						(1995)	
		Halbas		3		Joshi SH et al	
						(1990)	
		Gonds		13			
		Kawars		10.3			
		Oraons		8.5			
		Bhils		18.4			
		Bhilals		18.9			
		Barelas		17.7			
Tamil Nadu		Tribes of Kolli		1.86		Kalaivani V et al	
		Hills				(2001)	
Ladakh		Ladakhis		9.72		Dutta RN et al	
						(1975)	
Andhra		Lambada		5.2		Chandra M et al	
Pradesh						(2003)	
Arunachal		Tribal		8.5		Prasad SR et al	
Pradesh						(1983)	
Maharashtra		Raj Gond		28.03		Mukharjee M et al	
						(1990)	
		Kolam		14.15			
		Naik Gond		9.52			
		Pradhan		6.98			
Jharkhand		Paharia		2		Ghosh S et al	
						(2010)	
